# Volumetric Microsampling for Patient‐Centric Therapeutic Drug Monitoring in Clinical Pharmacology: A Scoping Review

**DOI:** 10.1002/jcph.70290

**Published:** 2026-09-04

**Authors:** Hari Prabhath Tummala, Abbie D. Leino, Nathaniel J. Rhodes, Kevin J. Downes, Marc H. Scheetz, Manjunath P. Pai

**Affiliations:** ^1^ Department of Clinical Pharmacy, College of Pharmacy University of Michigan Ann Arbor MI USA; ^2^ Division of Translational and Clinical Pharmacology Cincinnati Children's Hospital Medical Center Cincinnati OH USA; ^3^ Department of Pediatrics University of Cincinnati College of Medicine Cincinnati OH USA; ^4^ College of Pharmacy, Pharmacometrics Center of Excellence Midwestern University Downers Grove IL USA; ^5^ Department of Pediatrics Perelman School of Medicine at the University of Pennsylvania Philadelphia PA USA; ^6^ Division of Infectious Diseases Children's Hospital of Philadelphia Philadelphia PA USA

**Keywords:** model‐informed precision dosing, patient‐centric pharmacology, quantitative dried blood spot, volumetric absorptive microsampling, therapeutic drug monitoring

## Abstract

Accurate drug concentration measurement is essential for precision pharmacotherapy, but conventional therapeutic drug monitoring (TDM) requires venous sampling, increasing patient burden, and potentially limiting participation in TDM and model‐informed precision dosing (MIPD). Volumetric microsampling enables self‐collection of small, fixed volume capillary samples for centralized analysis and may reduce hematocrit‐related volumetric bias associated with conventional dried blood spots. This scoping review, conducted according to the PRISMA Extension for Scoping Reviews, synthesized 67 patient‐cohort studies published from 2014 through 2026. Studies spanned at least 18 countries and multiple clinical specialties. Liquid chromatography‐tandem mass spectrometry was used in 61 studies (91%), and most reported validation using or referencing EMA, FDA, IATDMCT, or CLSI criteria. Recovery was minimally affected across evaluated hematocrit ranges for most analytes, and many analytes remained stable in dried samples, although stability varied by analyte and storage conditions. Where assessed, patient preference was high (80%‐100%). However, interpretation against established plasma therapeutic ranges often required whole‐blood‐to‐plasma or capillary‐to‐venous conversion, with inconsistent derivation and validation methods. Fixed conversion factors performed well for lacosamide and levetiracetam but were less reliable for lamotrigine at high concentrations. Several analyte‐matrix combinations, including cefepime, vancomycin, meropenem, paclitaxel, abiraterone, and mitotane, did not consistently meet agreement criteria. Volumetric microsampling can support patient‐centered drug monitoring when validated for the specific analyte, matrix, device, and clinical application. Future research should prioritize drug‐specific optimization, multicenter validation, standardized conversion reporting, and integration with MIPD platforms.

## Introduction

Precision pharmacotherapy relies on accurate measurement of drug or active‐metabolite concentrations. Such measurements inform therapeutic drug monitoring (TDM) for dose adjustment and model‐informed precision dosing (MIPD) to achieve target concentrations. Although these approaches require more time and resources than population‐based dosing strategies, such as dosing by weight or body surface area, they are particularly valuable for drugs characterized by high pharmacokinetic variability, narrow therapeutic indices, or nonlinear dose–response relationships. Notable examples include calcineurin inhibitors, antiseizure medications, select antimicrobials, and certain oncology agents.^1^, ^2^, ^3^, ^4^

**Figure 1:**
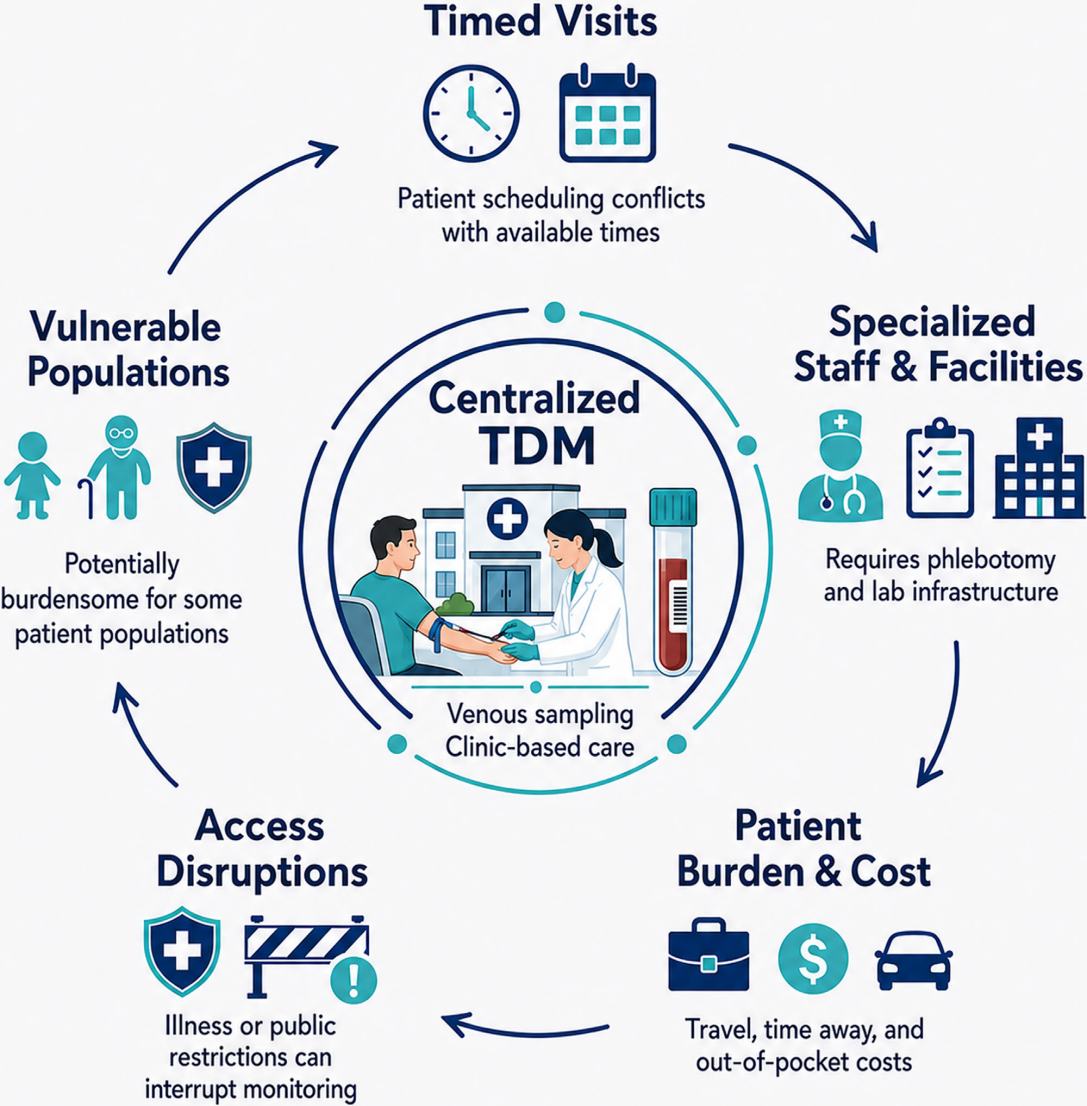
Schematic overview of the key challenges associated with centralized venous blood collection for therapeutic drug monitoring (TDM) and the patient‐centric advantages of volumetric microsampling.

Traditionally, TDM samples are obtained via venipuncture, a process that can present challenges for certain patient populations (Figure 1). Patients are often required to travel to clinical facilities at predetermined times, where trained personnel collect blood samples. These samples are either retained as whole blood or processed into plasma or serum, then stored at controlled temperatures until laboratory analysis. Each procedural step introduces potential variability and increases overall costs.^5^ For individuals with chronic illnesses requiring frequent TDM or MIPD, repeated venipuncture may result in anemia, vascular injury, and persistent needle‐related anxiety. These challenges are particularly pronounced among pediatric and geriatric populations, oncology patients, and individuals residing in rural areas.^6^

In the past two decades, several patient‐friendly alternatives to traditional blood draws have been developed, primarily utilizing capillary blood. Dried blood spot (DBS) sampling was among the earliest methods, requiring only a small blood volume and enabling samples to be shipped at ambient temperature. However, the accuracy of DBS can be compromised by the hematocrit effect, in which variations in blood viscosity influence analytical results.^7^, ^8^ Volumetric absorptive microsampling (VAMS), marketed as the Mitra device since 2014 (Neoteryx by Trajan), was designed to address this limitation by collecting a fixed volume of capillary blood, independent of typical hematocrit variation.^7^ Despite this advancement, results obtained from whole blood may still necessitate blood‐to‐plasma correction factors for accurate interpretation. Additional user‐friendly devices have also been introduced. Quantitative DBS systems, such as Capitainer qDBS, dispense precise blood volumes onto paper and can facilitate plasma separation if required. Self‐lancing devices, including those from the Tasso family, collect either liquid capillary blood or dried blood from the upper arm (Table 1). Devices such as HemaXis and HemaPEN have also entered the market, although they are relatively new and have not yet been extensively evaluated.^9^, ^10^, ^11^

**Table 1:** Comparison of Patient‐Centric Microsampling Devices Used for Therapeutic Drug Monitoring.

Feature	DBS	VAMS (Mitra)	qDBS (Capitainer)	Tasso
Sample volume	Variable; depends on hematocrit and spot size	Fixed 10/20/30 µL	Fixed 10 µL (gravimetric metering)	Appx. 150 µL plasma
Matrix	Whole blood (dried)	Whole blood (dried)	Whole blood (dried)	Plasma (separated on device)
Hematocrit effect	Significant (spot size, homogeneity)	Minimal across 0.20–0.55	Minimal (volumetric metering)	NA (plasma)
Self‐sampling	Possible; variable spot quality	Demonstrated, supervised and unsupervised	Demonstrated, supervised and unsupervised	Designed for unsupervised upper‐arm use
Ambient stability	Days to weeks (analyte dependent)	Up to 5 years for drugs like HCQ, MTX (analyte and storage condition specific)	Comparable to VAMS	Limited published data
Conversion to plasma	Hematocrit correction required	Conversion or B/P ratio required	Conversion required	Direct plasma, no conversion
Analytical platform	LC‐MS/MS; immunoassay	LC‐MS/MS predominant	LC‐MS/MS predominant	LC‐MS/MS predominant
Regulatory guidance	EMA, FDA, IATDMCT (legacy)	EMA, FDA, IATDMCT (current)	Emerging	Emerging
Key advantage	Simplicity, low cost	Hematocrit‐independent fixed volume	Volumetric accuracy + paper substrate	Plasma directly, no whole‐blood conversion
Key limitation	Hematocrit bias, sub‐punch error	Drug‐specific bias (ex: abiraterone)	Limited evidence base	Volume variability, few clinical studies

B/P, blood‐to‐plasma ratio; DBS, dried blood spot; EMA, European Medicines Agency; FDA, US Food and Drug Administration; HCQ, hydroxychloroquine; IATDMCT, International Association of Therapeutic Drug Monitoring and Clinical Toxicology; LC‐MS/MS, liquid chromatography‐tandem mass spectrometry; MTX, methotrexate; NA, not applicable; qDBS, quantitative dried blood spot; VAMS, volumetric absorptive microsampling.

Volumetric microsampling is currently employed for a wide range of drugs across diverse clinical contexts. Research has addressed both laboratory method development and large‐scale implementation, including scenarios in which patients self‐collect samples at home for laboratory analysis. To date, most reviews have concentrated on specific drug classes, such as immunosuppressants^11^ or antiseizure medications,^9^ or have emphasized technical performance. There remains a need for a comprehensive overview encompassing the full scope of clinical pharmacology.^8^, ^12^ As volumetric microsampling is increasingly applied to additional drug classes, including monoclonal antibodies, and in emerging fields such as neurology and oncology, an updated synthesis is warranted.

Therefore, this scoping review aims to achieve three primary objectives. The first objective was to map the application of patient‐oriented volumetric microsampling for TDM and MIPD within clinical pharmacology. The second was to summarize key validation parameters, including hematocrit effects, blood‐to‐plasma or capillary‐to‐venous result conversion, and sample logistics, which influence reproducibility. The third objective was to review evidence on patient‐centered outcomes, including acceptability, feasibility, adherence, and home sampling.

## Methods

This scoping review was conducted according to the Arksey and O'Malley framework, as refined by Levac et al,^13^, ^14^ and is reported in accordance with Preferred Reporting Items for Systematic reviews and Meta‐Analyses extension for Scoping Reviews (PRISMA‐ScR).^15^ A review protocol was retrospectively registered to open science framework post preparation of the manuscript.^16^

### Eligibility Criteria

Eligibility was determined using the Population Concept Context (PCC) framework. The population included human patients or healthy volunteers who provided biological samples for measurement of drug or active‐metabolite concentrations. The concept focused on patient‐oriented volumetric microsampling for TDM, defined as the use of devices that collect a fixed volume of capillary blood, or where applicable, saliva or capillary plasma, for quantitative determination of an exogenous therapeutic agent. Devices considered were VAMS‐format collectors (Mitra and Tasso‐M20), volumetric quantitative dried blood spot (qDBS) systems, and self‐lancing volumetric capillary collectors (Tasso+ for liquid plasma microsamples). The context encompassed clinical pharmacology practice, including outpatient, inpatient, home, and remote‐sampling settings. Peer‐reviewed articles published between January 2014 and February 2026 were included, corresponding to the period since the commercial introduction of VAMS for patient use.

Studies were included if they (a) employed a volumetric microsampling device as a primary or comparator sampling matrix, rather than solely dependent on conventional DBS or venipuncture; (b) measured drug or active‐metabolite concentrations in a real patient cohort; and (c) addressed at least one explicit patient‐centric parameter, such as home or remote sampling, inclusion of at‐risk populations, adherence assessment, sampling acceptability, or reduction of sampling burden. Studies were excluded if they were (a) limited to analytical method development without application to a real patient cohort; (b) reported only animal or in vitro investigations, focused on forensic toxicology, anti‐doping, or population biomarker screening rather than drug measurement; (c) used only microsampling formats outside the patient‐oriented volumetric scope; and (d) were narrative or systematic reviews, or re‐analyzed banked specimens without prospective patient sampling.

### Information Sources and Search Strategy

PubMed and Embase were used as the primary databases for this study. The search strategy combined controlled vocabulary and free‐text terms for the concept (“volumetric absorptive microsampling,” “VAMS,” “Mitra,” “Capitainer,” “Tasso,” and “qDBS”) and the topic (“therapeutic drug monitoring,” “TDM,” “drug quantification,” and “pharmacokinetics”). Outcome terms were omitted from the search to minimize identification bias and were instead applied during screening. The complete search strategy for both databases is provided in supplemental file 1.

### Selection of Sources of Evidence and Data Charting

**Figure 2:**
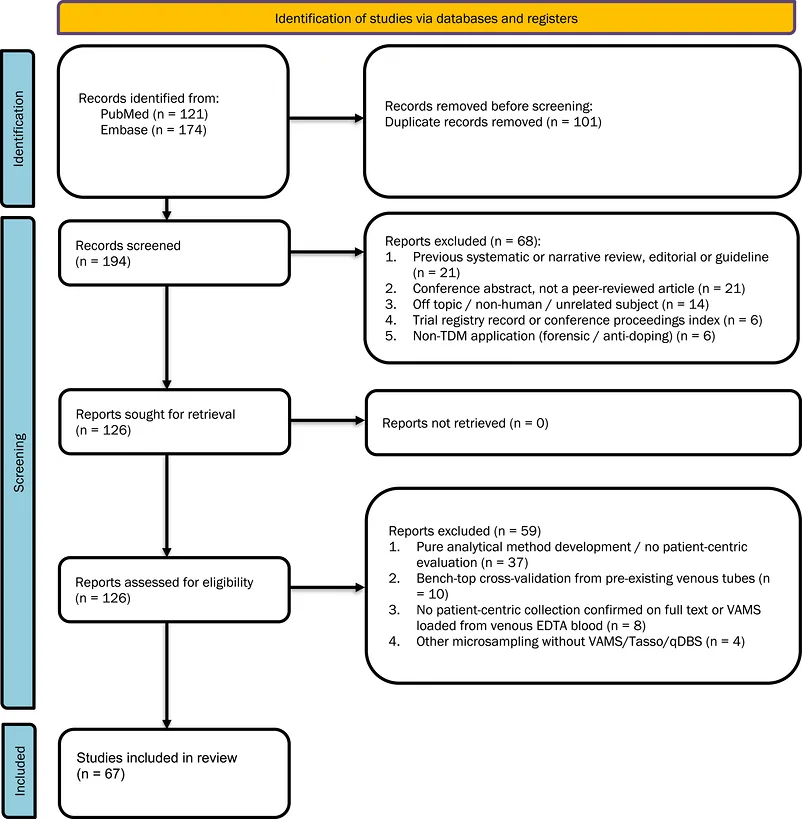
PRISMA‐ScR 2020 flow diagram for scoping reviews, with breakdown of records identified, screened, assessed, and included.

Records identified through the database search were exported to a reference manager. Titles and abstracts were screened against the eligibility criteria, followed by a full‐text review of records deemed potentially relevant. The PRISMA 2020 flow diagram (Figure 2) documents the selection process, including the total number of records identified, screened at the title/abstract stage, assessed at the full‐text stage, and included.

We developed a standardized data extraction form, which we piloted on five included studies. The variables collected were author, year, journal, study design, sampling device, drug and class, study population (including size, age range, and indication), patient‐centric features (such as home sampling, self‐sampling, acceptability, preference, and adherence), analytical platform, and the validation guidance referenced (European Medicines Agency [EMA] Bioanalytical Method Validation Guideline,^17^ US Food and Drug Administration [FDA] Bioanalytical Method Validation Guidance,^18^ International Association of Therapeutic Drug Monitoring and Clinical Toxicology [IATDMCT] Microsampling recommendations,^19^ Clinical and Laboratory Standards Institute [CLSI] EP09 method comparison guidance^20^). We also documented the approach to hematocrit, conversion strategies, key clinical validation findings (Passing–Bablok regression^21^ and Bland–Altman analysis^22^), the proportion of paired samples within EMA/IATDMCT acceptance windows, and stability claims. In this manuscript, “patient‐centric” refers to studies that specifically examine potential home or self‐sampling feasibility, acceptability, preferences, or adherence.

### Synthesis of Results

Due to the heterogeneity of analytes, devices, populations, and outcomes, we summarized the results narratively and organized them by therapeutic area. Consistent with the scoping review methodology, we did not perform quantitative data synthesis. Tables 2 and 3 present patient‐focused outcomes and quantitative validation metrics. During data extraction, we documented whether studies adhered to analytical validation guidance and included patient‐focused outcome measures. Large language models, including Claude Opus 4.7, assisted in verifying the selection process against the inclusion and exclusion criteria following manual filtering by the author. These models did not influence the primary study selection or the interpretation of results. ChatGPT 5.5 was used to generate Figure 1. All outputs from large language models were manually reviewed.

**Table 2:** Patient‐Centric and Key Clinical Findings of the Included Studies

Study (ref)	Drug(s)/Analyte(s)	Population (n)	Patient‐Centric Feature	Key Finding
Mbughuni et al, 2020^48^	Tacrolimus, cyclosporin A	47 to 49 paired transplant samples	Patient satisfaction, mailable home sampling	Capillary VAMS concordance versus venous WB: 79% (tacrolimus), 94% (cyclosporin A); 82% preferred capillary collection
Veenhof et al, 2020^49^	Tacrolimus	107 KTRs (130 paired)	Finger‐prick at home, mailed to lab	VAMS outperformed DBS versus venous WB
Kindem et al, 2021^60^	Tacrolimus	39 pediatric SOT (4 to 18 years)	Vulnerable group; finger‐prick	Cross‐validation acceptable (1/56 rejected)
Tron et al, 2021^39^	Tacrolimus	42 paired transplant samples	Capillary VAMS by nurse	Acceptable agreement versus venous reference
Marshall et al, 2020^55^	Tacrolimus, creatinine	131 renal transplant outpatient samples	Mailable; replaces repeat venipuncture	Validated versus routine LC‐MS/MS
Paniagua‐Gonzalez et al, 2022^59^	Tacrolimus, mycophenolic acid	SOT cohort	Self‐sampling alternative	VAMS met RCPA criteria for both analytes
Zwart et al, 2022^61^	Tacrolimus, mycophenolic acid, creatinine, iohexol	25 KTRs	Remote outpatient monitoring	Validated for simultaneous immunosuppressant and kidney function
Scuderi et al, 2023^53^	Tacrolimus, mycophenolic acid, mycophenolic acid glucuronide prednisolone	40 adult KTRs	Replaces venepuncture; multi‐analyte	Reliable simultaneous quantification of all four analytes (VAMS met FDA MAPE/MPPE acceptance)
Scuderi et al, 2023^54^	Tacrolimus, creatinine	40 adult KTR outpatients	Outpatient finger‐prick	Diagnostic‐test validation in routine outpatient cohort
Vethe et al, 2023^24^	Tacrolimus, creatinine, hemoglobin	25 (+12) KTRs	HCP versus supervised versus unsupervised self‐sampling	Self‐sampling acceptable with brief training
Yoo et al, 2023^57^	Everolimus	45 (32 analyzed) liver transplant	Outpatient post‐Tx monitoring	Good agreement; no conversion needed
Kocur et al, 2023^37^	Tacrolimus	50 pediatric KTRs	Vulnerable pediatric group	First Tac VAMS LC‐MS/MS in pediatric KTRs; >89% met EMA
Kocur et al, 2023^38^	Mycophenolic acid	50 pediatric KTRs	Finger‐stick capillary	86% to 88% met EMA; conversion model validated
Kocur et al, 2024^50^	Sirolimus	Pediatric KTRs	Vulnerable group; capillary	76% met IATDMCT; clinical applicability shown
Kocur et al, 2024^51^	Ciclosporin	Pediatric KTRs	Home‐based, remote therapy	Validated; addresses adherence in pediatrics
Leino et al, 2024^27^	Tacrolimus, mycophenolic acid	25 KTRs (50 paired)	Patient preference and usability	Both devices validated; upper arm preferred
Nierychlewski et al, 2025^56^	Tacrolimus, cyclosporin A, kynurenine, tryptophan, creatinine	26 anonymized transplant samples	Capillary alongside venous	Multimarker offline‐SPE LC‐MS/MS validated
Drevland et al, 2025^58^	Mycophenolic acid and mycophenolic acid glucuronide	12 KTRs (69 paired)	Finger‐prick AUC prediction	Conversion model enables limited‐sampling AUC
Kocur et al, 2025^25^	Ganciclovir	Pediatric KTRs	Home self‐sampling feasibility	Both devices clinically validated
Kocur et al, 2025^26^	Everolimus	33 adult SOT (66 matched)	Decentralized outpatient monitoring	First Mitra‐Capitainer comparison for everolimus
Kocur et al, 2025^52^	Mycophenolic acid and mycophenolic acid glucuronide (saliva)	Pediatric KTRs	Non‐invasive saliva collection	Saliva VAMS strongly correlated with wet saliva (SRCC 0.990); saliva concentrations correlated poorly with plasma—saliva is not a reliable substitute for plasma TDM of mycophenolic acid and mycophenolic acid glucuronide
Juan et al, 2026^44^	Mycophenolic acid, tacrolimus	Adult lung + renal transplant (paired)	Less‐invasive AUC scenarios	Mycophenolic acid formula A: 86% within ±20% of plasma; tacrolimus required no Hct correction; Hct‐adjusted conversion improved predictive performance
Vethe et al, 2019^62^	Tacrolimus	27 renal transplant recipients (679 paired)	Mailed home samples; full dose interval	Mean difference −3.1% versus venous whole blood; <8% outside ±20%; results unaffected by postal shipment
Gustavsen et al, 2020^63^	Tacrolimus	27 renal transplant recipients (53 profiles)	Patient self‐sampling; AUC without hospital stay	Microsamples at 0, 1, and 3 h, predicted venous AUC_0–12_; C_0_ alone was inadequate
Zhao et al, 2025^47^	Mycophenolic acid, mycophenolic acid glucuronide	10 pediatric lupus nephritis patients	Finger‐prick; 3 timed samples; home testing	Hct‐adjusted capillary VAMS matched plasma; AUC correlated with plasma AUC (R^2^ = 0.97)
Friedl et al, 2019^30^	Mitotane	ACC patients on mitotane	Home capillary collection	First minimally invasive HPLC‐UV VAMS method for mitotane in adrenocortical carcinoma; clinical correlation with plasma poor ‐ limited value without method‐specific target range
Krutzmann et al, 2023^66^	Imatinib	Home self‐sampling by patients; Morisky–Green adherence questionnaire	Home + Morisky–Green adherence	33 CML patients self‐collected VAMS at home; Morisky–Green adherence captured; method validated against bench plasma (no paired HCP‐vs‐self analytical comparison)
Radovanovic et al, 2023^69^	Paclitaxel	Outpatient clinic capillary collection by nursing staff	Capillary self‐sampling	Approximately 50% of capillary samples outside ±20% agreement with venous plasma (12/20 paired; only 5/13 within ±20%); VAMS and plasma considered non‐interchangeable
Isberner et al, 2022^70^	Dabrafenib, trametinib	27 BRAF‐V600 melanoma (18 micro)	At‐home capillary self	95.3% correct self‐sampling rate
Zimmermann et al, 2023^71^	Nilotinib, cabozantinib, dabrafenib, trametinib, ruxolitinib	59 cancer patients (591 samples)	At‐home acceptance + sample quality	Clinical validation across 5 TKIs
Jacobs et al, 2024^72^	Abemaciclib, anastrozole, exemestane, letrozole, palbociclib, ribociclib, tamoxifen, endoxifen	25 BC patient samples	Adherence monitoring of oral endocrine therapy	VAMS not interchangeable with plasma; multi‐matrix LC‐HRMS
Maggadani et al, 2021^73^	Tamoxifen, endoxifen, 4‐hydroxytamoxifen, N‐desmethyltamoxifen	30 ER+ breast cancer patients	Finger‐prick; ambient‐temperature storage	Endoxifen below the 3.9 ng/mL dried blood threshold in 2 patients; stable 30 days at room temperature
Meertens et al, 2024^40^, ^64^	Abiraterone, alectinib, cabozantinib, imatinib, olaparib, sunitinib, and metabolites	Outpatient clinic samples	Outpatient and at‐home intent	Analytical validation; abiraterone failed acceptance
Meertens et al, 2025^64^	Abiraterone, alectinib, cabozantinib, imatinib, olaparib, sunitinib, and metabolites	153 cancer patients	Home‐sampling feasibility	Samples collected properly in 70% of cases; at least 1 valid sample analyzed in 91% of patients (153 patients)
Shafiei et al, 2024^68^	Capecitabine and metabolites	10 adult cancer patients	Patient preference + pain (VAS)	100% preferred microsampling
Verougstraete et al, 2025^65^	Dasatinib, imatinib	Hospital capillary VAMS by trained personnel; home implementation discussed as next step	Home capillary collection	Acceptable agreement versus plasma/WB
Chi et al, 2025^74^	Trastuzumab, bevacizumab, pembrolizumab	Cancer patients	At‐home mAb TDM	First VAMS LC‐MS for therapeutic mAbs
Velghe et al, 2020^79^	Antiepileptic drugs	263 children (Uganda + DRC)	Remote rural Africa; pediatric	Therapeutic 52.9% VPA, 21.4% CBZ, 65.7% PB; remainder subtherapeutic; VAMS and DBS fit‐for‐purpose in low‐resource settings
Dubois et al, 2020^77^	Cannabidiol (Epidiolex)	5 refractory epilepsy (Dravet syndrome)	Pediatric vulnerable group	Strong agreement capillary versus venous
Pigliasco et al, 2020^42^	Cannabidiol, THC	5 pediatric Epidiolex	Pediatric vulnerable group	Online‐prep UHPLC‐MS/MS validated
Palmisani et al, 2023^78^	Perampanel (saliva)	Adult epilepsy patients	Domiciliary non‐invasive	Salivary VAMS validated to EMA criteria
Pigliasco et al, 2024^76^	Cenobamate	10 adult cenobamate‐treated PWE	Adult epilepsy + polypharmacy	First VAMS method for cenobamate
Moorthy et al, 2019^87^	Cannabinoids (THC, CBD, CBN)	Pediatric PK study	Patient‐centric framing; vulnerable group	Patient‐centric VAMS method developed and applied in pediatric PK
Cancellerini et al, 2024^75^	13 antiseizure medications	301 PWE (464 measurements)	Self‐collection at home	Largest unsupervised self‐sampled VAMS study
Klimpel et al, 2021^45^	Lacosamide, lamotrigine, levetiracetam	72 adult inpatients with epilepsy	Fingertip sampling replaces venepuncture	Serum/capillary ratios 1.14 (LCM), 0.85 (LTG), 1.22 (LEV); simple ratio and Passing–Bablok performed equally
Hagemann et al, 2023^46^	Lacosamide, lamotrigine, levetiracetam	59 adult inpatients with epilepsy	Two sampling times; independent validation cohort	Simple ratio best; EMA criterion met for LCM and LEV, and for LTG only at ≤11 µg/mL
Mardhiani et al, 2023^34^	Phenytoin	30 epilepsy patients	Finger‐prick; HPLC where LC‐MS/MS unavailable	21 of 30 patients subtherapeutic; VAMS stable 31 days at room temperature
Cancellerini et al, 2025^23^	5 antiseizure medications	30 PWE	Home finger‐prick; vulnerable group	Capitainer qDBS validated versus plasma
Barco et al, 2017^80^	Piperacillin‐tazobactam, meropenem, linezolid, ceftazidime	Pediatric patients	PK/PD + TDM in pediatrics	Multiplex LC‐MS/MS validated
Moorthy et al, 2020^41^	Cefepime	Pediatric PK study	Pediatric vulnerable group	Method validated in pediatric clinical study
Moorthy et al, 2020^81^	Vancomycin	Pediatric clinical study	Pediatric vulnerable group	Method developed and applied clinically
Ramadan et al, 2023^83^	Meropenem	Critically ill PICU children	Vulnerable PICU group	VAMS systematically underestimated meropenem
Lazaridi et al, 2025^84^	Amoxicillin, metronidazole, azithromycin	12 volunteers (6 healthy, 6 periodontitis)	Patient + dentist acceptability	Concentration evolution captured for all three
Grando et al, 2025^28^	Amikacin, gentamicin, tobramycin	23 paired patient samples	Self‐lancing capillary plasma	Amikacin and gentamicin clinically quantified in patient plasma microsamples; tobramycin validated analytically only
Zimbelmann et al, 2025^29^	Fluconazole	49 pediatric post‐HCT (211 samples)	Pediatric antifungal prophylaxis	Capillary captures concentrations at low Hct
Kocur et al, 2026^82^	Cefazolin	Pilot pediatric ICU cohort	Vulnerable; limited blood volume	Robust LC‐MS/MS across blood, plasma, ultrafiltrate
Rendati et al, 2024^33^	Rifampicin, isoniazid	21 tuberculosis patients	Finger‐prick; low‐resource setting	52% subtherapeutic for ≥1 drug; only 29% in range for both; HPLC method fit for purpose
Marasca et al, 2020^32^	Sertraline, fluoxetine, citalopram, vortioxetine	Antidepressant outpatients	Self/home‐sampling enabled	HPLC‐UV‐FL VAMS + oral fluid method validated for outpatient antidepressant TDM
Marasca et al, 2022^31^	Clozapine (and N‐desmethyl‐, N‐oxide metabolites)	5 psychiatric patients	Schizophrenia compliance	Novel b‐VAMS, p‐VAMS, and mfDBS approaches coupled to HPLC‐ED applied to clozapine TDM
Millan‐Santiago et al, 2023^85^	Cariprazine	Psychiatric outpatients on cariprazine	Schizophrenia compliance	First VAMS TDM for cariprazine
Jacobs et al, 2021^86^	13 antipsychotics	10 patients (17 drug intakes) with matched plasma	Finger‐prick; adherence monitoring	Adherence classification agreed with plasma in 16/17 intakes; 5 drugs degraded >15% after 1 week at 24°C
Tanna et al, 2018^88^	Atenolol, lisinopril, simvastatin, valsartan	Adherent versus non‐adherent volunteers	Cardiovascular adherence	VAMS‐HRAM screen distinguishes adherence patterns
Jacobs et al, 2021^95^	10 antihypertensive drugs	26 hypertensive patients (55 intakes)	Acceptability questionnaire; home‐sampling intent	Dose‐related concentrations used as cut‐offs; VAMS and plasma not interchangeable; good patient acceptance
Qu et al, 2017^89^	Hydroxychloroquine + metabolites	RA patients	Home capillary collection	Capillary VAMS validated for hydroxychloroquine in RA; sample collection in the patient's home setting flagged as a future possibility
Brady et al, 2021^35^	Hydroxychloroquine, methotrexate	RA (85) and SLE (126)	Chronic‐disease compliance + biobanking	Capillary hydroxychloroquine and methotrexate measured on VAMS stable up to 5 years at −80°C storage (ambient stability <1 year)
Marahatta et al, 2016^90^	Hydroxyurea	Sickle‐cell‐anemia patients	Pediatric SCA compliance	Stable‐isotope HPLC‐ESI‐MS/MS validated; accuracy 97% to 112%
Soenen et al, 2022^91^	Adalimumab	7 psoriasis (SUPRA‐A sub study)	Home VAMS + perception questionnaire	First home‐VAMS biologic TDM sub study.

ACC, adrenocortical carcinoma; AUC, area under the curve; BC, breast cancer; b‐VAMS, whole‐blood volumetric absorptive microsampling; CBZ, carbamazepine; CML, chronic myeloid leukemia; DBS, dried blood spot; DRC, Democratic Republic of Congo; EMA, European Medicines Agency; FDA, US Food and Drug Administration; HCP, healthcare personnel; HCT, hematopoietic‐cell transplant; Hct, hematocrit; HPLC‐ED, high‐performance liquid chromatography with electrochemical detection; HPLC‐UV, high‐performance liquid chromatography with ultraviolet detection; HPLC‐UV‐FL, high‐performance liquid chromatography with ultraviolet and fluorescence detection; HRAM, high‐resolution accurate mass; KTRs, kidney transplant recipients; mAbs, monoclonal antibodies; MAPE, median absolute percentage prediction error; mfDBS, microfluidic‐generated dried blood spot; MPPE, median percentage prediction error; PB, phenobarbital; PICU, pediatric intensive care unit; PK, pharmacokinetics; p‐VAMS, plasma volumetric absorptive microsampling; PWE, persons with epilepsy; RA, rheumatoid arthritis; SCA, sickle‐cell anemia; SLE, systemic lupus erythematosus; SOT, solid‐organ transplant; SRCC, Spearman rank correlation coefficient; TDM, therapeutic drug monitoring; TKI, tyrosine kinase inhibitor; VAMS, volumetric absorptive microsampling; VAS, visual‐analog scale; VPA, valproic acid; WB, whole blood.

NR denotes data not reported.

**Table 3:** Quantitative Validation Metrics Across Volumetric Microsampling Studies

Study	Analyte	Ref. Matrix^a^	Slope (95% CI)^b^	Intercept (95% CI)^b^	Mean Bias (95% CI or 95% LOA)^c^	r/R^2^/ICC (95% CI)
Mbughuni et al, 2020^48^	Cyclosporin A (cap‐MSD vs vWB)	vWB	0.95 (0.84, 1.05)	13.95 (−3.95, 23.94)	−9.85 ng/mL (23.7, −43.42)	0.88*
	Tacrolimus (cap‐MSD vs vWB)	vWB	0.98 (0.84, 1.11)	0.99 (−0.15, 1.83)	−0.88 ng/mL (1.64, −3.40)	0.82*
Vethe et al, 2019^62^	Tacrolimus (VAMS mailed)	vWB	1.012	−0.5869	−3.1% (−3.9, −2.3)	0.975
	Tacrolimus (VAMS direct to laboratory)	vWB	1.006	−0.5735	−4.2% (−5.1, −3.4)	0.976
	Tacrolimus (VAMS mailed, C12 and C13)	vWB	0.987	−0.3365	−6.0% (−7.7, −4.3)	0.926
Veenhof et al, 2020^49^	Tacrolimus	vWB	0.88 (0.81 to 0.97)	0.01 (−0.47, 0.39)	95% LoA: 0.78 to 1.22	NR
Kindem, 2021^60^	Tacrolimus	vWB	0.97 (0.85 to 1.05)	0.047 (−0.37, 0.66)	NR	NR
Tron et al, 2021^39^	Tacrolimus	vWB	0.98 (0.91, 1.11)	−0.2 (−0.99, 0.18)	−0.4 ng/mL (−0.08, 0.92)	0.97
Marshall et al, 2020^55^	Creatinine	vS	0.91	1.81	−6.5% (−8.5, 4.5%)	NR
	Tacrolimus	vWB	0.93	0.07	−5.6% (−8.5, 2.7%)	NR
Paniagua‐González et al, 2022^59^	Mycophenolic acid	vP	1.92 (1.64, 2.07)	−63.57 (−165.31, 47.5)	NR	0.98^
	Tacrolimus	vWB	1.38 (1.16, 1.54)	−0.07 (−0.68, 0.5)	NR	0.86^
Zwart et al, 2022^61^	Creatinine mean concentration	vP	1.17 (0.92, 1.71)	−2.85 (−9.74, 0.93)	0.98 (95% LoA 0.76 to 1.20)^#^	NR
	Iohexol concentration (conversion corrected)	vP	1.01 (0.91, 1.11)	−2.37 (−15.8, 14.8)	1.00 (95% LoA 0.64 to 1.36)^#^	NR
	Iohexol mGFR (conversion corrected)	vP	1.39 (0.82, 2.08)	−20.5 (−66.1, 8.02)	1.02 (95% LoA 0.63 to 1.40)^#^	NR
	Mycophenolic acid AUC_0–12_ (conversion corrected)	vP	1.11 (0.69, 1.35)	−.67 (−13.9, 13.8)	1.05 (95% LoA 0.76 to 1.33)^#^	NR
	Mycophenolic acid concentration (conversion corrected)	vP	1.07 (0.97, 1.13)	0.00 (−0.21, 0.18)	1.07 (95% LoA 0.77 to 1.37)^#^	NR
	Tacrolimus AUC_0–24_	vWB	1.12 (0.92, 1.32)	−4.69 (−34.3, 16.7)	1.06 (95% LoA 0.83 to 1.30)^#^	NR
	Tacrolimus C_0_	vWB	1.09 (0.88, 1.55)	−0.06 (−2.31, 0.85)	1.07 (95% LoA 0.84 to 1.31)^#^	NR
	Tacrolimus concentration	vWB	1.05 (0.98, 1.14)	0.14 (−0.37, 0.65)	1.07 (95% LoA 0.78 to 1.35)^#^	NR
	eGFR (VAMS derived)	eGFR (vCr)	1.10 (0.88, 1.33)	−4.12 (−15.9, 5.77)	1.04 (95% LoA 0.76 to 1.33)^#^	NR
Zhao et al, 2025^47^	Mycophenolic acid (capillary VAMS, Hct adjusted)	vP	1.10 (0.93 to 1.20)	0.14 (−0.18, 0.39)	NR	0.975
	Mycophenolic acid (capillary VAMS, unadjusted)	vP	1.68 (1.47 to 1.95)	0.16 (−0.14, 0.33)	NR	0.971
	Mycophenolic acid (venous VAMS, Hct adjusted)	vP	1.02 (0.91 to 1.14)	0.002 (−0.32, 0.26)	NR	0.986
	Mycophenolic acid (capillary vs venous VAMS)	vVAMS	1.01 (0.92 to 1.17)	0.09 (−0.09, 0.20)	−0.24 µg/mL (−2.34, 1.90)	0.962
Scuderi et al, 2023^53^	Mycophenolic acid glucuronide	vP	1.0 (0.9, 1.1)	0.1 (−3.9, 1.4)	1.6% (95% LoA −9.6 to 32.8)	NR
	Mycophenolic acid	vP	1.0 (0.9, 1.0)	0.01 (0)	−5.3% (95% LoA −38.6 to 27.9)	NR
	Prednisolone	vP	1.0 (0.9, 1.1)	−2.5 (−20.1, 32.1)	2.5% (95% LoA −51.1 to 56.2)	NR
	Tacrolimus	vWB	1.1 (1.0, 1.1)	−0.6 (−1.0, 0.3)	−1.6% (95% LoA −21.7 to 18.4)	NR
Scuderi et al, 2023^54^	Creatinine	vS	0.65 (0.57 to 0.74)	NR	1.42% (95% LoA −34.1 to 36.9)	NR
	Tacrolimus	vWB	1.08 (1.03 to 1.13)	NR	−1.6% (95% LoA −21.7 to 18.4)	NR
Vethe et al, 2023^24^	Creatinine (Capitainer, HCP)	vP	0.83	5.0	0.6% ± 4.9% (−1.5 to 2.6)	0.994*
	Creatinine (Capitainer, self‐supervised)	vP	0.83	5.0	1.4% ± 7.2% (−1.6 to 4.4)	NR
	Creatinine (Capitainer, self‐alone)	vP	0.83	5.0	0.0% ± 6.0% (−2.4 to 2.5)	NR
	Creatinine (Mitra, HCP)	vP	0.93	4.4	0.8% ± 7.3% (−2.2 to 3.9)	0.991*
	Creatinine (Mitra, self‐supervised)	vP	0.93	4.4	2.5% ± 10.8% (−2.0 to 7.0)	NR
	Creatinine (Mitra, self‐alone)	vP	0.93	4.4	0.8% ± 12.2% (−4.4 to 6.0)	NR
	Tacrolimus (Capitainer, HCP)	vWB	NR	NR	−2.6% ± 10.1% (−6.9 to 1.6)	NR
	Tacrolimus (Capitainer, self‐supervised)	vWB	NR	NR	−4.3% ± 12.2% (−9.5 to 0.9)	NR
	Tacrolimus (Capitainer, self‐alone)	vWB	NR	NR	−5.9% ± 10.3% (−10.8 to 0.9)	NR
	Tacrolimus (Mitra, HCP)	vWB	NR	NR	−3.7% ± 14.1% (−9.8 to 2.4)	NR
	Tacrolimus (Mitra, self‐supervised)	vWB	NR	NR	−0.4% ± 15.2% (−6.9 to 6.2)	NR
	Tacrolimus (Mitra, self‐alone)	vWB	NR	NR	−1.5% ± 12.8% (−8.6 to 5.6)	NR
Yoo et al, 2023^57^	Everolimus	vWB	NR	NR	−0.79 ng/mL ± 0.90 ng/mL (95% LoA: 2.55 to 0.97)	0.92*
Kocur, 2023^37^	Tacrolimus (vs CMIA)	vWB (CMIA)	0.948	0.934	−0.80 ng/mL (−1.22 to 0.38)	0.92 (0.8676 to 0.9558)
	Tacrolimus (vs WB‐LC‐MS/MS)	vWB	0.9129	1.006	0.1046 ng/mL (−0.37 to 0.57)	0.91 (0.8342 to 0.9440)
Kocur, 2023^38^	Mycophenolic acid	vP	1.00 (0.92 to 1.09)	−0.13 (−0.35 to 0.03)	+10.90% (5.24 to 16.56)	NR
	Mycophenolic acid (independent validation, n = 30)^‡^	vP	0.98 (range 0.80 to 1.29)	−0.03 (range −0.54 to 0.06)	NR	0.99 (0.96 to 0.99)
Kocur, 2024^50^	Sirolimus (vs WB‐LC‐MS/MS)	vWB	1.01 (0.95 to 1.08)	0.18 (−0.07 to 0.50)	−3.30%	0.9892 (0.9664 to 0.9972)* 0.9893 (0.9826 to 0.9934)^
Kocur, 2024^51^	Cyclosporin A (vs WB‐LC‐MS/MS)	vWB	0.98	−1.16	−1.68% (−5.14 to 1.78)	0.99 (0.964 to 0.998)
Leino et al, 2024^27^	Mycophenolic acid (Tasso, correction ×1.3)	vS	1.09 (0.94, 1.23)	−138.89 (−325.53, 79.24)	82 (−116.74, 280.84); 95% LOA: (1288.88, 1452.98)	NR
	Mycophenolic acid (Tasso, individual Hct correction)	vS	0.99 (0.86, 1.10)	−126.95 (−313.10, 37.26)	−218.18 (−383.69, −52.67); 95% LOA: (1359.61, 923.25)	NR
	Mycophenolic acid (Tasso, mean VAMS‐to‐serum ratio = 1.31)	vS	1.08 (0.93, 1.23)	−138.89 (−339.32, 67.87)	62.24 (−135.84, 260.31); 95% LOA: (1303.78, 1428.25)	NR
	Mycophenolic acid (finger, correction x1.47)	vS	1.07 (0.97, 1.16)	−101.33 (−266.37, 39.20)	−4.80 (−176.05, 166.45); 95% LOA: (1198.18, 1188.58)	NR
	Mycophenolic acid (finger, individual Hct correction)	vS	1.11 (0.99, 1.15)	−115.21 (−251.28, 14.81)	29.34 (−109.61, 168.30); 95% LOA: (939.02, 997.71)	NR
	Mycophenolic acid (finger, mean VAMS‐to‐serum ratio = 1.47)	vS	1.07 (0.97, 1.16)	−101.33 (−266.37, 39.20)	−4.80 (−176.05, 166.45); 95% LOA: (1198.18, 1188.58)	NR
	Mycophenolic acid (uncorrected, fingerstick Mitra vs serum)	vS	1.59 (1.47, 1.72)	−131.05 (−289.90, −0.65)	836.80 ng/mL (593.31, 1080.30); 95% LOA −860.02, 2533.62	0.79 (0.70, 0.85)**
	Mycophenolic acid (uncorrected, upper‐arm Tasso‐M20 vs serum)	vS	1.42 (1.23, 1.64)	−141.5 (−347.18, 69.60)	676.50 ng/mL (425.87, 927.13); 95% LOA −1051.97, 2404.97	0.82 (0.74, 0.87)**
	Tacrolimus (Mitra, finger)	vWB	1.0 (0.92, 1.11)	0.3 (−0.73, 1.21)	0.63 ng/mL (0.16, 1.10); 95% LOA −2.62, 3.88	0.95 (0.92, 0.97)**
	Tacrolimus (Tasso, upper arm)	vWB	0.94 (0.86, 1.01)	0.50 (−0.36, 1.59)	−0.1 ng/mL (−0.64, 0.45); 95% LOA −3.86, 3.66	0.96 (0.93, 0.97)**
Nierychlewski et al, 2025^56^	Creatinine	vS	0.88	20.021	−15.23%	0.995*
	Cyclosporin A	vWB	0.934	8.558	2.45%	0.982*
	Tacrolimus	vWB	1.02	−0.577	−4.49%	0.932*
Drevland, 2025^58^	Mycophenolic acid	vP	1.02	−0.33	1.9% (−4.4% to 8.1%)	0.96
	Mycophenolic acid glucuronide	vP	0.99	−1.16	2.7% (−1.2% to 6.6%)	0.97
Kocur, 2025^25^	Ganciclovir (plasma vs VAMS)	vP	1.0180 (0.9682 to 1.0642)	−0.0344 (−0.0811 to 0.0073)	−4.59% (−7.50 to 1.69)	0.987 (0.977 to 0.993)^
	Ganciclovir (plasma vs qDBS)	vP	1.0068 (0.9597 to 1.0425)	−0.0337 (−0.0779 to 0.0074)	−5.49% (−8.16 to 2.83)	0.991 (0.985 to 0.995)^
	Ganciclovir (VAMS vs qDBS)	qDBS	1.0090 (0.9784 to 1.0382)	−0.0097 (−0.0283 to 0.0095)	0.90% (−0.68 to 2.48)	0.995 (0.991 to 0.997)^
	Ganciclovir (WB vs qDBS)	vWB	1.0063 (0.9631 to 1.0459)	0.0146 (−0.0215 to 0.0565)	3.70% (1.62 to 5.77)	0.988 (0.979 to 0.993)^
	Ganciclovir (WB vs VAMS)	vWB	1.0160 (0.9717 to 1.0672)	0.0200 (−0.0215 to 0.0537)	4.59% (2.24 to 6.94)	0.990 (0.983 to 0.995)^
Kocur, 2025^26^	Everolimus (WB vs qDBS)	vWB	0.938 (0.852 to 1.071)	0.1435 (−0.460 to 0.634)	−1.73% (−6.48 to 3.02)	0.932*
	Everolimus (WB vs VAMS)	vWB	1.017 (0.923 to 1.127)	−0.040 (−0.603 to 0.382)	−0.68% (−4.89 to 3.53)	0.935*
Kocur et al, 2025^52^	Mycophenolic acid	Saliva	1.0047 (0.9686, 1.0473)	0.1656 (−0.1122, 0.7092)	3.30% (0.62 to 5.97); LoA −23.12% to 29.72%	0.990 (0.9860 to 0.9940)
	Mycophenolic acid glucuronide	Saliva	1.0315 (0.9897, 1.0721)	0.9495 (−0.0159, 1.9748)	6.49% (3.49 to 9.49); LoA −28.13% to 36.11%	0.981 (0.9720 to 0.9870)
Juan, 2026^44^	Mycophenolic acid—Formula A‐fix (Hct = 0.45) VAMS versus plasma	vP	0.79 (0.74 to 0.83)	−28.13 (−50.33 to 7.38)	30.59% (LOA −24.78% to 85.96%)	NR
	Mycophenolic acid—Formula A‐individual VAMS versus plasma	vP	0.98 (0.94 to 1.02)	−14.64 (−29.00 to 15.87)	3.08% (LOA −33.56% to 39.73%)	NR
	Mycophenolic acid—Formula B (Wenkui‐style) VAMS versus plasma	vP	1.01 (0.98 to 1.06)	−16.11 (−27.08 to 7.15)	4.15% (LOA −35.17% to 43.47%)	NR
	Mycophenolic acid—Formula C (regression with Hct as covariate) VAMS versus plasma	vP	0.84 (0.78 to 0.89)	155.71 (95.93 to 180.95)	−68.55% (LOA −397.33% to 260.62%)	NR
	Mycophenolic acid—Formula D (Kromdijk DBS derived) VAMS versus plasma	vP	0.98 (0.94 to 1.01)	−17.74 (−30.40 to 9.13)	3.80% (LOA −33.71% to 41.31%)	NR
Radovanovic et al, 2023^69^	Paclitaxel (capillary Mitra vs venous Mitra)	v‐Mitra	0.8569 (0.3924 to 1.096)	NR	NR	NR
	Paclitaxel (measured vs estimated plasma from WB, Cf)	vP	0.9495 (0.8533 to 1.061)	NR	0.78% (95% LoA −25.78% to 27.35%)	NR
	Paclitaxel (measured vs estimated plasma from WB, Eq)	vP	1.000 (0.9039 to 1.120)	NR	4.43% (95% LoA −19.9% to 28.8%)	NR
	Paclitaxel (plasma vs estimated from venous Mitra, Cf)	vP	1.106 (0.868 to 1.521)	NR	NR	NR
	Paclitaxel (plasma vs estimated from venous Mitra, Eq)	vP	0.963 (0.713to 1.31)	NR	NR	NR
	Paclitaxel (venous Mitra vs plasma direct)	vP	1.193 (0.930 to 1.61)	−11.37	NR	NR
	Paclitaxel (venous WB vs plasma)	vP	0.8433 (0.7608 to 0.9432)	NR	NR	NR
Zimmermann et al, 2023^71^	Cabozantinib	vS	1.848 (1.664 to 2.008)	−0.057 (−70.603 to 36.167)	5.1% (−2.9 to 13.1) ^##^	0.9627
	Dabrafenib	vS	1.785 (1.538 to 2.122)	0.414 (−5.559 to 4.759)	1.6% (−5.3 to 8.4) ^##^	0.9674
	Nilotinib	vS	1.478 (1.246 to 1.635)	150.874 (56.961 to 242.819)	7.5% (−4.3 to 19.4) ^##^	0.9427
	Ruxolitinib	vS	0.715 (0.657 to 0.826)	0.110 (−2.600 to 3.021)	3.0%^##^	0.9466
	Trametinib	vS	0.453 (0.329 to 0.616)	−2.713 (−7.452 to 1.280)	0.5%^##^	0.5811
Meertens et al, 2025^64^	Abiraterone	vP	1.11 (1.02, 1.28)	2.90 (−0.76, 6.83)	NR	0.953
	Alectinib	vP	0.71 (−0.41, 1.06)	282.3 (−457, 931)	NR	0.761
	Alectinib‐M4	vP	0.51 (0.36, 0.70)	5.09 (−59.1, 67.2)	NR	0.779
	Cabozantinib	vP	1.57 (1.14, 1.88)	−116 (−246, 106)	NR	0.932
	D4A	vP	1.08 (0.99, 1.40)	0.21 (−0.23, 0.36)	NR	0.946
	Imatinib	vP	1.35 (1.00, 1.84)	−168 (−705, 150)	NR	0.794
	N‐desethyl sunitinib	vP	0.68 (0.57, 0.80)	0.48 (−1.72, 3.86)	NR	0.921
	N‐desmethyl imatinib	vP	1.56 (1.37, 1.74)	−80.0 (−124, 235.6)	NR	0.954
	Olaparib	vP	1.34 (1.03, 1.72)	−41.6 (−811, 1044)	NR	0.913
	Sunitinib	vP	0.50 (0.41, 0.58)	1.24 (−1.67, 5.32)	NR	0.967
Shafiei et al, 2024^68^	5‐deoxy‐5‐fluorocytidine	vP	1.183 (0.508 to 1.448)	437.05 (−299.18 to 1287.86)	NR	0.88; 0.775*
	5‐deoxy‐5‐fluorouridine	vP	0.663 (0.364 to 1.00)	−22.166 (−1197.615 to 580.129)	NR	0.902; 0.814*
	5‐fluorouracil	vP	0.749 (0.345 to 1.774)	−8.645 (−134 to 56)	NR	0.89; 0.69*
	5‐fluorouracil	vVAMS	NR	NR	NR	0.90; 0.83*
	Capecitabine (capillary VAMS)	vP	0.810	0.862	28% (4.02 to 52.00)	0.973; 0.94*
	Capecitabine (capillary VAMS)	vVAMS	1.021 (0.605 to 1.395)	30.75 (−956.67 to 1181.03)	NR	0.935; 0.94*
Verougstraete et al, 2025^65^	Dasatinib (calculated plasma)	vP	NR	NR	7.51% (1.11% to 13.9%)	NR
	Dasatinib (capillary VAMS)	vWB	NR	NR	1.42% (−3.87% to 6.80%)	NR
	Dasatinib (capillary VAMS)	vVAMS	NR	NR	−5.38% (−9.25% to 1.52%)	NR
	Dasatinib (venous VAMS)	vWB	NR	NR	6.77% (3.33% to 10.2%)	NR
	Imatinib (capillary VAMS)	vWB	NR	NR	−12.1% (−16.2% to 8.15%)	NR
	Imatinib (capillary VAMS)	vVAMS	NR	NR	−0.15% (−3.65% to 3.34%)	NR
	Imatinib (venous VAMS)	vWB	NR	NR	−12.0% (−14.65 to 9.41%)	NR
Chi, 2025^74^	Bevacizumab^§^	vP	29.9997	−0.0732	NR	0.99
	Pembrolizumab^§^	vP	8.5410	−0.4341	NR	0.99
	Trastuzumab	vP	5.3699	−0.2272	NR	0.98
Velghe et al, 2020^79^	Carbamazepine	DBS	1.40 (1.16 to 1.64)	−0.81 (−1.58 to 0.09)	NR	NR
	Valproic acid	DBS	1.23 (0.90 to 1.55)	−4.88 (−22.0 to 8.0)	NR	NR
Dubois, 2020^77^	Cannabidiol (cVAMS)	vP	NR	NR	NR	0.9325*
Pigliasco et al, 2024^76^	Cenobamate	vP	0.72 (−1.55 to 2.91)	2.24 (0.65 to 1.16)	NR	NR
Cancellerini, 2024^75^	Carbamazepine^†^	vP	0.72 (0.57 to 0.93)	2.09 (0.82 to 3.28)	0.12 (−3.26 to 3.51)	NR
	Carbamazepine ^‡^	vP	0.80 (0.64 to 0.98)	1.99 (−0.64 to 3.21)	−0.11 (−3.29 to 3.07)	NR
	Lacosamide ^†^	vP	0.68 (0.49 to 0.88)	1.14 (0.48 to 2.19)	0.03 (−3.33 to 3.40)	NR
	Lacosamide ^‡^	vP	0.70 (0.47 to 0.90)	1.18 (0.09 to 2.55)	0.04 (−2.84 to 2.92)	NR
	Levetiracetam ^†^	vP	1.04 (0.88 to 1.21)	0.26 (−1.90 to 1.97)	−0.68 (−7.60 to 6.23)	NR
	Levetiracetam ^‡^	vP	1.04 (0.88 to 1.17)	0.15 (−1.49 to 1.34)	−0.35 (−6.20 to 5.50)	NR
	Lamotrigine ^†^	vP	1.05 (0.92 to 1.17)	−0.15 (−0.55 0.40)	−0.25 (−3.61 to 3.12)	NR
	Lamotrigine ^‡^	vP	0.99 (0.89 to 1.09)	0.31 (−0.17 to 0.84)	−0.14 (−3.16 to 2.88)	NR
	Oxcarbazepine ^†^	vP	0.99 (0.78 to 1.34)	0.44 (−5.00 to 3.53)	−0.24 (−6.91 to 6.43)	NR
	Oxcarbazepine ^‡^	vP	0.96 (0.74 to 1.18)	1.13 (−1.92 to 4.58)	−0.10 (−7.05 to 6.85)	NR
	Phenobarbital ^†^	vP	0.84 (0.57 to 1.12)	0.94 (−2.05 to 3.52)	0.34 (−5.87 to 6.56)	NR
	Phenobarbital ^‡^	vP	0.87 (0.56 to 1.11)	1.14 (−1.06 to 3.73)	0.26 (−6.35 to 6.87)	NR
	Phenytoin ^†^	vP	0.52 (0.38 to 1.06)	3.45 (0.16 to 4.54)	0.38 (−5.63 to 6.39)	NR
	Phenytoin ^‡^	vP	0.66 (0.45 to 0.92)	2.86 (1.36 to 4.55)	−0.45 (−4.58 to 5.48)	NR
	Perampanel ^†^	vP	0.92 (0.64 to 1.21)	19.53 (−28.73 to 54.60)	1.92 (−103.98 to 107.81)	NR
	Perampanel ^‡^	vP	0.82 (0.51 to 1.13)	32.14 (−2.60 to 73.51)	2.39 (−112.52 to 117.29)	NR
	Topiramate ^†^	vP	1.91 (1.39 to 2.93)	−5.12 (−12.49 to 0.98)	−1.09 (−5.91 to 3.73)	NR
	Topiramate ^‡^	vP	2.22 (1.03 to 4.25)	−6.387 (−18.65 to 0.71)	−1.25 (−8.07 to 5.57)	NR
	Valproic acid ^†^	vP	1.40 (0.99 to 1.88)	−12.74 (−36.40 to 10.10)	−6.49 (−65.03 to 52.06)	NR
	Valproic acid ^‡^	vP	1.43 (0.99 to 1.86)	−16.18 (−43.58 to 8.93)	−6.86 (−66.46 to 52.75)	NR
	Zonisamide ^†^	vP	1.89 (1.42 to 2.89)	−22.06 (−48.51 to 9.82)	−3.78 (−23.75 to 16.18)	NR
	Zonisamide ^‡^	vP	1.75 (1.33 to 2.53)	18.18 (6.94 to 41.36)	v3.32 (−21.23 to 14.60)	NR
Klimpel et al, 2021^45^	Lacosamide	vS	1.07 (0.95 to 1.18)	0.10 (−0.17, 0.72)	1.14 (1.05 to 1.24)	0.96
	Lamotrigine	vS	0.75 (0.70 to 0.80)	0.46 (0.14, 0.77)	0.85 (0.80 to 0.91)	0.95
	Levetiracetam	vS	1.26 (1.11 to 1.46)	−0.88 (−2.85, 1.36)	1.22 (1.15 to 1.29)	0.95
Hagemann et al, 2023^46^	Lacosamide	vS	NR	NR	0.67 (0.39 to 0.94)	0.93^
	Lamotrigine	vS	NR	NR	0.60 (−0.12 to 1.31)	0.91^
	Levetiracetam	vS	NR	NR	0.95 (0.21 to 1.69)	0.97^
Cancellerini, 2025^23^	Carbamazepine	vP	0.7802 (−0.05490 to 1.6153)	1.193 (−5.7158 to 8.1017)	0.24 (−3.18 to 1.98)	NR
	Lacosamide (qDBS)	vP	0.8456 (0.6033 to 1.0878)	0.6444 (−0.7447 to 2.0335)	0.38 (−1.24 to 0.86)	NR
	Levetiracetam	vP	0.9610 (0.7663 to 1.1556)	1.7543 (−0.7457 to 4.2543)	0.04 (−1.60 to 4.28)	NR
	Lamotrigine	vP	1.2731 (0.8785 to 1.6678)	−0.5993 (−2.8910 to 1.6923)	0.03 (−1.58 to 3.37)	NR
Ramadan et al, 2023^83^	Meropenem (VAMS WB vs plasma, raw)	vP	NR	NR	29.1% (95% LoA: 18.3, +36.4%)	NR
Grando, 2025^28^	Amikacin—corrected (×1.101 multiplier)	vP	0.9735 (0.9143 to 1.1166)	−0.02019 (−1.6287 to 1.8443)	−0.12% (LOA −21.12% to 20.8%)	NR
	Amikacin—uncorrected venous versus capillary	vP	0.8768 (0.8214 to 1.0061)	−0.01517 (−1.4773 to 1.7714)	10.1% (LOA −11.4% to 31.6%)	0.98
	Gentamicin—venous versus capillary plasma	vP	1.0385 (0.7500 to 1.3947)	−0.3135 (−0.9921 to 0.7250)	−1.1% (LOA −42.3% to 40.1%)	0.979
Zimbelmann et al, 2025^29^	Fluconazole (EDTA‐WB Mitra)	vP (Mitra)	0.81 (0.54 to 0.92)	3.16 (0.02 to 7.10)	−0.74% (95% LoA: −37.5, 36.0)	NR
	Fluconazole (EDTA‐WB Mitra)	vS (Mitra)	1.01 (0.83 to 1.13)	−0.09 (−3.77 to 2.63)	1.33% (95% LoA: −31.6, 34.2)	0.99*
Marasca et al, 2020 (OF‐VAMS)^32^	All antidepressants (Sertraline, Fluoxetine, Citalopram, Vortioxetine)	OF	1.0000 (0.9135, 1.0746) (pooled OF‐VAMS versus fluid OF)	0.2500 ng/mL (−1.5500, 2.7596)	NR	0.9941*
Marasca et al, 2020^32^	Citalopram + metabolites DCT/DDC	vP	1.0054 (0.9652, 1.0388)	0.2512 ng/mL (−1.3305, 1.5911)	NR	0.9964*
	Fluoxetine + metabolite norfluoxetine	vP	1.0054 (0.9652, 1.0388)	0.2512 ng/mL (−1.3305, 1.5911)	NR	0.9964*
	Sertraline + metabolite NSR	vP	1.0054 (0.9652, 1.0388)	0.2512 ng/mL (−1.3305, 1.5911)	NR	0.9964*
	Vortioxetine	vP	1.0054 (0.9652, 1.0388)	0.2512 ng/mL (−1.3305, 1.5911)	NR	0.9964*
Brady, 2021^35^	hydroxychloroquine (5‐year Cohort)	vWB	NR	NR	−7.1% (−12.6 to 1.6)	0.96^
	hydroxychloroquine (3‐year Cohort)	vWB	NR	NR	−2.9% (−6.0 to 0.2)	0.94^
	Methotrexate‐PG3	RBC methotrexate‐PG_3_	NR	NR	−2.9% (−9.0 to 3.1)	0.91^
Soenen et al, 2022^91^	Adalimumab (capillary VAMS)	vS	NR	NR	2.7 µg/mL (1.96 to 3.35)	0.87
	Adalimumab (capillary VAMS)	vVAMS	NR	NR	1.30 µg/mL (0.72 to 1.87)	0.91

AUC, area under the concentration–time curve; Cf, conversion factor; D4A, Δ4‐abiraterone; Eq, regression equation; Hct, hematocrit; LC‐MS/MS, liquid chromatography‐tandem mass spectrometry; MSD, microsampling device (Mitra in Mbughuni 2020); NSR, norsertraline; SD, standard deviation; Tasso‐M20, Tasso upper‐arm VAMS device; VAMS, volumetric absorptive microsampling; WB, whole blood.

Values are reported verbatim from the source publications; NR indicate the metric was not reported. Multiple analytical scenarios from the same study (sample collector, conversion strategy, comparator matrix, and device) appear as separate rows.

^a^ Ref. Matrix: vWB, venous whole blood; vP, venous plasma; vS, serum; vVAMS, in‐clinic venous‐blood VAMS comparator; v‐Mitra, venous Mitra (VAMS‐loaded); OF, oral fluid; Saliva, wet saliva; DBS, dried blood spot; qDBS, quantitative dried blood spot (Capitainer); CMIA, chemiluminescent microparticle immunoassay; RBC MTX‐PG_3_, red‐blood‐cell methotrexate polyglutamate‐3; eGFR (vCr), estimated glomerular filtration rate from venous creatinine.

^b^ Slope and intercept from Passing–Bablok regression (PB) unless the cell labels otherwise.

^ᶜ^ Mean bias from Bland–Altman analysis with 95 % CI of the bias or 95 % LOA / LoA (limits of agreement). r denotes Pearson correlation. ICC, intraclass correlation.

^†^ Patient self‐collected.

^‡^ Regression corrected or nurse collected.

^§^ Alternate correction (Hct based / fixed factor).

* R^2^.

^^^ ICC.

^#^ Mean ratio.

^##^ MPPE (median percentage prediction error).

## Results

### Characteristics of Included Studies

A total of 295 records were identified in PubMed and Embase using our search strategy. After removing 101 duplicates, 194 unique records were screened. Of these, 67 met all eligibility criteria and were included (Figure 2). The included studies were published between 2014 and February 2026. Notably, there was an increase in publications toward the end of the study period, with 26 of 67 studies (39%) published in 2024 or later. The research originated from at least 18 countries across Europe, North America, Asia, Africa, and South America.

The Mitra‐format VAMS device was the most frequently used, appearing in 57 of 67 studies. Capitainer qDBS was included in four studies, either alone or as a comparator.^23^, ^24^, ^25^, ^26^ Tasso devices, which are self‐lancing collectors for dried whole blood or liquid capillary plasma, were used in two studies focused on TDM for immunosuppressants^27^ and aminoglycosides.^28^ Several studies directly compared two or more devices to evaluate usability and analytical performance under similar clinical conditions.^24^, ^25^, ^27^ Liquid chromatography‐tandem mass spectrometry (LC‐MS/MS) or ultra‐high‐performance liquid chromatography‐tandem mass spectrometry (UHPLC‐MS/MS) was employed in 61 of 67 studies (91%). Exceptions included HPLC‐UV methods for fluconazole, mitotane, antidepressants, and clozapine, as reported by Zimbelmann et al,^29^ Friedl et al,^30^ and Marasca et al.^31^, ^32^ HPLC with photodiode array or ultraviolet detection was also used for rifampicin and isoniazid in tuberculosis and for phenytoin in epilepsy, in both cases because LC‐MS/MS was not routinely available locally.^33^, ^34^ Sample volumes were typically 10, 20, or 30 µL per device. The evaluated analytical stability of the dried tip was approximately 14 days for many analytes and up to 5 years for hydroxychloroquine and methotrexate at certain storage conditions (−80°C), as demonstrated by Brady et al.^35^ The studies encompassed a wide range of patient populations, with a frequent focus on vulnerable groups. Pediatric cohorts were included in 18 studies and adult outpatients in 39 studies. Some studies also addressed rare or stigmatizing conditions, such as nodding syndrome, Dravet syndrome, sickle‐cell anemia, refractory psychiatric illness, pediatric lupus nephritis, and adrenocortical carcinoma.

### Analytical Validation and Methodological Landscape

Volumetric microsampling has shown consistent progress since its introduction in TDM. Method validation in published studies typically relied on one or more of four principal frameworks: the EMA Bioanalytical Method Validation Guideline,^17^ the FDA Bioanalytical Method Validation Guidance,^18^ the IATDMCT recommendations for microsampling validation,^19^, ^36^ and the CLSI EP09 method‐comparison guidance.^20^ Studies following EMA or FDA standards generally required both accuracy and precision to be within 15%, or 20% at the lower limit of quantification. Most studies reported that results met or exceeded the IATDMCT's 20% acceptance threshold when compared to venipuncture‐based reference methods, frequently using Passing–Bablok regression and Bland–Altman analysis (Table 3). The increasing application of CLSI's EP09 guidance, which is more common in clinical chemistry than in TDM, suggests that laboratory medicine is adapting to patient‐centered sampling.

Hematocrit demonstrated minimal impact on VAMS recovery for most analytes across a broad range, from approximately 20% to 55%.^7^, ^11^ This represents a significant advantage of volumetric devices over traditional DBS methods. Researchers have specifically evaluated pediatric and oncology hematocrit ranges for drugs such as tacrolimus,^37^ mycophenolic acid,^38^ fluconazole following allogeneic hematopoietic‐cell transplantation,^29^ and tacrolimus in adult solid‐organ transplants.^39^ In all instances, results satisfied EMA acceptance criteria at varying hematocrit levels. Only a limited number of analytes, including abiraterone, failed to meet these criteria at certain hematocrit values.^40^ Cefepime is frequently cited as an example where recovery is hematocrit dependent,^40^, ^41^ underscoring the necessity of evaluating each drug individually rather than applying a universal rule. It is also essential to distinguish between the requirement for a hematocrit‐aware conversion factor to translate whole blood to plasma and device hematocrit dependence, which qDBS and VAMS were specifically designed to minimize.

Sample preparation predominantly involved solvent extraction with stable‐isotope‐labeled internal standards. Samples were dried prior to extraction, and multiple studies demonstrated that sufficient drying time is necessary for consistent results. Most studies systematically reported matrix effects and extraction recovery. Automation has become increasingly prevalent, as illustrated by an online workflow for preparing cannabidiol samples in capillary blood^42^ and a high‐throughput automated tacrolimus VAMS pipeline developed by Carling et al, which facilitated routine implementation.^10^ A persistent challenge is the conversion of VAMS whole‐blood concentrations to plasma concentrations suitable for clinical use based on the preponderance of existing evidence applying plasma concentration to clinical care, or alternatively, establishing whole‐blood target ranges for certain analytes to avoid conversion. Researchers have employed three primary approaches: correction factors based on the blood‐to‐plasma partition ratio,^43^ Passing–Bablok regression with paired patient samples, and direct plasma measurement using devices such as Tasso+.^28^ Tacrolimus is an exception, as it is measured in whole blood and does not require conversion, indicating that the appropriate method is drug dependent. A recent study by Juan et al compared conversion strategies for mycophenolic acid and found that well‐designed correction factors can maintain bias within 10% compared to plasma.^44^ Two related epilepsy studies underscore the necessity of validating conversion factors in independent cohorts. Klimpel et al reported average serum‐to‐capillary‐blood ratios of 1.14 for lacosamide, 0.85 for lamotrigine, and 1.22 for levetiracetam in 72 adults.^45^ Subsequently, Hagemann et al evaluated these factors and demonstrated superior performance compared to regression‐based approaches in 59 patients. For lamotrigine, this criterion was met only at capillary concentrations of 11 µg/mL or lower.^46^ For mycophenolic acid, it was determined that hematocrit adjustment eliminated the systematic difference between capillary VAMS and plasma.^47^ These findings underscore the importance of clearly reporting conversion methods rather than adopting a universal approach.^19^

### Therapeutic‐Area Applications

#### Transplantation and Immunosuppression

Transplant patients constitute the largest and most extensively studied population for volumetric microsampling. One of the earliest large‐scale clinical studies, conducted by Mbughuni et al, utilized VAMS to monitor tacrolimus and cyclosporin A in two cohorts of 50 transplant patients, with 49 and 47 paired capillary and venous samples, respectively.^48^ For VAMS samples derived from venous blood, concordance with standard assays was 89% for tacrolimus and 98% for cyclosporin A. In capillary blood, concordance was 79% for tacrolimus and 94% for cyclosporin A. Notably, 82% of patients expressed a preference for the capillary collection method.^48^ Another study involving 107 patients demonstrated that both DBS and VAMS methods were clinically acceptable after adjustment (DBS 96.6% vs VAMS 83.0% within ±15%), although VAMS exhibited a higher sample rejection rate (32.3% vs 6.2%). The authors proposed that VAMS could serve as a replacement for venous blood sampling in tacrolimus monitoring.^49^

A comprehensive comparison by Vethe et al evaluated the Mitra and Capitainer qDBS devices for quantifying tacrolimus, creatinine, and hemoglobin in 25 kidney transplant patients, with an additional follow‐up cohort of 12 patients.^24^ For tacrolimus and predicted area under the curve (AUC), 79%‐96% of Capitainer samples and 77%‐95% of Mitra samples fell within ±20% of reference values. Capitainer demonstrated consistent performance across various sampling scenarios, including samples collected by healthcare professionals (72%‐88%) and those collected by patients, either under supervision or independently (92%‐96%). In contrast, the success rate for Mitra declined when patients performed self‐collection (52%‐72%). These findings underscore the critical role of usability, in addition to analytical accuracy, in self‐sampling initiatives. In a separate cohort of 25 kidney transplant patients, both the Tasso‐M20 upper‐arm device and the Mitra finger‐prick device were validated. Patient surveys indicated a strong preference for the upper‐arm device regarding comfort and ease of use.^27^

Multiple studies involving pediatric kidney transplant recipients have validated the use of VAMS coupled with LC‐MS/MS for drugs such as tacrolimus,^37^ mycophenolic acid,^38^ sirolimus,^50^ cyclosporin A,^51^ and ganciclovir,^25^ as well as for measuring mycophenolic acid in saliva.^52^ In these investigations, 70%‐90% of paired samples satisfied EMA acceptance criteria (Table 3). Comparable validation studies have been conducted in adult outpatient cohorts. Scuderi et al demonstrated that finger‐prick VAMS is suitable for monitoring tacrolimus, mycophenolic acid, and prednisolone,^53^ in addition to paired serum creatinine assessments.^54^ Validation of tacrolimus and creatinine testing using the Mitra device was achieved in 131 routine kidney transplant outpatient samples, with sample stability at room temperature supporting their suitability for postal transport.^55^ Further research has broadened the scope of monitoring to include tryptophan metabolites and kidney function markers via offline solid‐phase extraction LC‐MS/MS.^56^ Feasibility studies have also been conducted for everolimus monitoring in liver transplant recipients.^57^ Recent advancements include a finger‐prick VAMS method for mycophenolic acid and its glucuronide metabolite, with AUC prediction modeling in 12 kidney transplant patients,^58^ a direct comparison of DBS and VAMS for tacrolimus and mycophenolic acid using RCPA criteria,^59^ a cross‐validation study in 39 pediatric patients aged 4 to 18,^60^ and validation of capillary VAMS for tacrolimus in paired transplant samples.^39^ Remote monitoring utilizing mailed devices has also been implemented to assess immunosuppressant levels and kidney function in 25 outpatient kidney transplant patients.^61^ Vethe et al established the foundation for this method. In a study involving 27 kidney transplant patients, capillary microsamples sent by mail differed from venous blood by an average of ‐3.1%. Fewer than 8% of sample pairs were outside ±20%, and the dried samples remained stable for 1 month at room temperature.^62^ The approach was subsequently expanded from monitoring trough levels to estimating overall drug exposure, specifically predicting the 12‐h tacrolimus AUC using microsamples collected at 0, 1, and 3 h after dosing.^63^ Moreover, Zhao et al further utilized VAMS with three timed samples to optimize mycophenolate mofetil dosing in 10 children with lupus nephritis. In this study, capillary concentrations adjusted for hematocrit closely matched plasma AUC (R^2^ = 0.97).^47^ Collectively, transplantation offers the most comprehensive and consistent evidence base for volumetric microsampling, encompassing a wide range of devices, pharmacological agents, and patient populations.

#### Oncology

Oral targeted cancer therapies have transitioned treatment into patients homes, often necessitating prolonged administration over several months or years. This extended dosing schedule is compatible with the benefits of home‐based microsampling, as evidenced by recent studies. The largest oncology study to date, conducted by Meertens et al, included 153 outpatients receiving abiraterone, alectinib, cabozantinib, imatinib, olaparib, or sunitinib, with VAMS performed in both clinical and home settings.^64^ The investigators applied conversion factors to translate whole‐blood VAMS results into plasma concentrations, finding that seven out of 10 drugs and metabolites met the ICH M10 acceptance criteria. However, abiraterone, its active metabolite D4A, and alectinib did not meet these standards, indicating that further optimization is required for certain analytes.^40^, ^64^ Verougstraete et al extended this methodology to chronic myeloid leukemia in 53 patients (33 receiving dasatinib and 20 receiving imatinib), reporting strong concordance between VAMS and plasma measurements for imatinib in a controlled environment, which supports the potential for future home‐based monitoring.^65^ Furthermore, Krutzmann et al combined microsampling with the Morisky–Green adherence scale, demonstrating that objective drug quantification can complement patient‐reported adherence assessments and enhance the detection of non‐adherence.^66^

Although cytotoxic chemotherapy is monitored at home less frequently than oral targeted therapies, it remains an important focus in microsampling research. One VAMS method for 5‐fluorouracil, capecitabine, and their metabolites demonstrated stability for up to 9 months.^67^ In a small pilot study involving 10 patients taking capecitabine, researchers used Likert and visual‐analog scales to confirm patient preference for the method and reduced pain.^68^ Capillary self‐sampling was also evaluated for paclitaxel TDM; however, early results indicated high variability and did not support clinical implementation, as half of the samples were outside acceptable limits.^69^ For BRAF‐mutant melanoma, dabrafenib and trametinib have been monitored in both serum and self‐collected capillary blood, with at‐home VAMS shown to be feasible in 18 patients.^70^ Another study validated VAMS in 59 cancer patients (591 samples) for nilotinib, cabozantinib, dabrafenib, trametinib, and ruxolitinib, and included assessments of at‐home sample quality and stability.^71^ This work built upon earlier laboratory studies mapping 10 kinase inhibitors and their VAMS‐to‐plasma ratios.^43^

Two recent studies are particularly significant in this field. Jacobs et al demonstrated that adherence to seven oral endocrine therapies for breast cancer (abemaciclib, anastrozole, exemestane, letrozole, palbociclib, ribociclib, tamoxifen, and the endoxifen metabolite) can be monitored using liquid chromatography‐high‐resolution mass spectrometry (LC‐HRMS) across four sample types, including VAMS, in 25 matched patient samples.^72^ This methodology addresses a critical gap in objective adherence measurement for hormone receptor‐positive cancers. VAMS was earlier applied to the same treatment setting, quantifying tamoxifen, endoxifen, 4‐hydroxytamoxifen, and N‐desmethyltamoxifen in 30 Indonesian patients with estrogen receptor‐positive breast cancer. Endoxifen fell below the 3.9 ng/mL dried blood threshold for therapeutic failure in two patients, showing that microsampling can flag candidates for dose escalation.^72^, ^73^ In another important study, Chi et al introduced a VAMS LC‐MS/MS method for monitoring therapeutic monoclonal antibodies (trastuzumab, bevacizumab, and pembrolizumab), thereby expanding microsampling applications to therapeutic‐protein TDM, which has previously relied primarily on ELISA with plasma.^74^ For mitotane, the only approved therapy for adrenocortical carcinoma, Friedl et al developed an HPLC‐UV VAMS method for home monitoring. However, this technique is not yet suitable for routine clinical use, as VAMS and plasma concentrations did not correlate sufficiently, indicating that further analytical refinement is necessary for certain analytes.^30^ In summary, oncology is a rapidly expanding yet heterogeneous field for microsampling, with the strongest evidence supporting oral targeted therapies and adherence monitoring, and emerging applications in chemotherapy and monoclonal antibody therapies.

### Neurology and Antiseizure Medications

Epilepsy represents a strong use case for patient‐centered TDM, as many patients require long‐term antiseizure therapy, face adherence challenges, or live in regions where access to specialty care is limited. The largest study of patient‐self‐collected microsampling to date, conducted by Cancellerini et al, enrolled 301 persons with epilepsy who provided 464 finger‐prick VAMS measurements covering 13 antiseizure medications under real‐world conditions.^75^ The same Italian program subsequently validated a Capitainer qDBS panel for five antiseizure medications in 30 patients in an ambulatory setting, providing a foundation for future home‐based collection.^23^ A VAMS‐based LC‐MS/MS method for the third‐generation antiseizure medication cenobamate was validated in 10 epilepsy patients with refractory focal‐onset seizures, supporting the transition to TDM for newer‐generation agents.^76^

Cannabidiol (Epidiolex) TDM in pediatric refractory epilepsy was first demonstrated by Dubois et al in patients with Dravet syndrome, who showed strong agreement between capillary VAMS and venous reference samples.^77^ An independent finger‐prick VAMS method for cannabidiol and tetrahydrocannabinol using online sample preparation was applied to five pediatric patients receiving Epidiolex.^42^ A novel salivary VAMS technique for perampanel, developed by Palmisani et al, enabled a non‐invasive monitoring of an analyte previously dependent on plasma sampling, illustrating how alternative biological matrices can further reduce patient burden.^78^ A globally important contribution by Velghe et al applied VAMS and DBS‐based microsampling for antiepileptic‐drug monitoring in 263 children (68 with nodding syndrome in Uganda, 58 with epilepsy in Uganda, and 137 with onchocerciasis‐associated epilepsy in the Democratic Republic of the Congo). The work demonstrated feasibility in decentralized settings where venipuncture, cold‐chain logistics, and laboratory access are limited.^79^ Klimpel et al collected paired venous serum and capillary VAMS samples from 72 inpatients and derived conversion factors for lacosamide, lamotrigine, and levetiracetam, thereby addressing a key barrier to routine clinical implementation.^45^ Validation was performed in 59 patients sampled both before and 2 h after the morning dose, confirming accuracy across the clinically relevant concentration range for lacosamide and levetiracetam.^46^ These two studies are among the few to both derive and independently validate a conversion factor, which aligns with recommended procedures for microsampling assays. Older antiepileptic agents have also been investigated, with an HPLC‐UV VAMS method for phenytoin applied to 30 Indonesian epilepsy patients; 21 of these patients were below the therapeutic range and would require dose adjustment.^34^ Collectively, epilepsy is one of the strongest patient‐centered use cases for volumetric microsampling, supported by large real‐world cohorts, pediatric applications, newer antiseizure agents, alternative matrices, and feasibility in decentralized settings.

### Infectious Disease

Antimicrobial TDM by microsampling is gaining traction, particularly in pediatric and immunocompromised patients where blood volume is constrained. A multiplex VAMS LC‐MS/MS assay for piperacillin‐tazobactam, meropenem, linezolid, and ceftazidime was developed for pediatric patients,^80^ followed by separate cefepime^41^ and vancomycin^81^ assays validated in pediatric clinical trials. Fluconazole TDM was specifically targeted in 49 pediatric post‐hematopoietic‐cell‐transplant patients across 211 samples, providing evidence that whole‐blood concentrations can be reliably determined from microsamples at clinically relevant hematocrit values, though clinical validation in actual capillary blood remains a future step.^29^ A 2026 contribution describes robust LC‐MS/MS quantification of cefazolin across whole blood, plasma, and plasma ultrafiltrate, showing method portability across matrices.^82^ Aminoglycosides (amikacin, gentamicin, and tobramycin) have been quantified in capillary plasma microsamples using the Tasso+ device, which delivers a liquid rather than dried matrix.^28^ An important cautionary signal comes from a pediatric ICU study by Ramadan et al, which observed an inconsistent and unpredictable correlation between whole‐blood VAMS and plasma concentrations for meropenem, concluding that not every antimicrobial is suited to whole‐blood microsampling.^83^ A real‐world pilot study in dental pharmacotherapy evaluated capillary VAMS antibiotic monitoring (amoxicillin, metronidazole, and azithromycin) in 12 volunteers with and without periodontitis and noted some drug and time‐dependent differences between capillary VAMS and venous plasma.^84^ Tuberculosis is a further use case, given long treatment durations and frequent subtherapeutic exposure. Rendati et al measured rifampicin and isoniazid on VAMS by HPLC at 2 and 6 h post‐dose in 21 Indonesian patients and found that 52% were subtherapeutic for at least one drug while only 29% were within range for both.^33^ These studies suggest that antimicrobial microsampling is a promising but still analyte and matrix‐dependent application, with strong pediatric rationale. Expanding assay coverage is tempered by evidence that some antimicrobials require further clinical validation before routine implementation.

### Psychiatry, Cardiovascular, Rheumatology, Endocrine, and Other Indications

Volumetric microsampling has also expanded beyond high‐volume areas into specialties where adherence assessment, decentralized monitoring, or sampling of vulnerable populations matter most. In psychiatry, whole‐blood and oral‐fluid VAMS have been validated for sertraline, fluoxetine, citalopram, and vortioxetine in outpatient antidepressant therapy.^32^ A follow‐up extended to clozapine TDM in psychiatric patients, evaluating whole‐blood VAMS alongside a microfluidic‐generated DBS approach, benchmarked against reference plasma.^31^ Cariprazine, an atypical antipsychotic with limited TDM literature, was validated and successfully applied to clinical samples from a small proof‐of‐concept group of three psychiatric patients.^85^ Jacobs et al broadened this work to adherence monitoring, validating a finger‐prick VAMS LC‐HRMS/MS method for the 13 most frequently prescribed antipsychotics at variable hematocrit values and applying it to 17 drug intakes with matched plasma samples. Adherence classification agreed between the two matrices in all but one case, although five of the drugs degraded by more than 15% after 1 week in the dried tip at 24°C.^86^ A cannabinoid VAMS method for pediatric pharmacokinetic studies has also been reported.^87^ For cardiovascular adherence monitoring, Tanna et al coupled VAMS with high‐resolution accurate‐mass mass spectrometry to monitor atenolol, lisinopril, simvastatin, and valsartan in adherent and non‐adherent volunteers, illustrating how high‐resolution mass spectrometry adds analytical headroom (providing high selectivity via a narrow 5 ppm mass extraction window) for multi‐drug screens.^88^

In rheumatology, Brady et al demonstrated that capillary VAMS samples of hydroxychloroquine and methotrexate remained stable for up to 5 years at ‐80C storage in patients with rheumatoid arthritis and systemic lupus erythematosus sampled across two clinical studies (n = 85 in the 2015 study and n = 126 in the 2017 study).^35^ An earlier validation of capillary VAMS for hydroxychloroquine and metabolites in 54 RA patients utilized in‐clinic finger‐prick sampling, which the authors noted successfully demonstrated the feasibility of transitioning to the patient's home setting.^89^ In hematology and hemoglobinopathies, Marahatta et al quantified hydroxyurea in sickle‐cell‐anemia patients to support pediatric adherence monitoring.^90^ Biologic TDM, historically limited to ELISA on plasma, was advanced by Soenen et al in the home‐VAMS substudy of the SUPRA‐A trial, which measured adalimumab in psoriasis patients alongside patient‐perception questionnaires.^91^ That study set the stage for the broader monoclonal antibody application by Chi et al described above. These smaller specialty applications show that volumetric microsampling is most valuable when it solves a specific clinical barrier, including adherence assessment, biologic monitoring, decentralized sampling, or reduced blood‐volume burden in vulnerable populations.

### Patient‐Centric Outcomes and Home‐Sampling Feasibility

A notable characteristic of this body of literature is the explicit evaluation of humanistic outcomes in addition to analytical performance. Patient acceptability or preference was directly assessed in 15 of 67 included studies (22%). Although this proportion is modest, it highlights the ongoing need for comprehensive humanistic outcome reporting. Where measured, patient preference for microsampling has been consistently strong. For example, Mbughuni et al found that 82% of transplant patients preferred capillary VAMS over venipuncture.^48^ In capecitabine‐treated cancer patients, a 100% preference for microsampling was documented using Likert and visual‐analog‐scale instruments.^68^ Patient‐experience assessments comparing devices in kidney transplant recipients indicated a preference for upper‐arm Tasso collection over finger‐prick.^27^ Objective concentration measurements for adherence assessment have been incorporated into chronic‐disease management in cardiovascular medicine,^88^ chronic myeloid leukemia,^66^ and oral endocrine breast cancer therapy^72^ and may be useful to address the limitations of self‐reported adherence.

The possible feasibility of home self‐sampling was evaluated in 28 studies. The most rigorous assessment was a three‐arm comparison by Vethe et al, which examined healthcare personnel, supervised self‐sampling, and unsupervised self‐sampling and demonstrated acceptable performance under unsupervised conditions following brief patient training.^24^ Real‐world implementation in resource‐limited settings has been documented in rural Uganda and the Democratic Republic of Congo.^79^ Collectively, these patient‐centric outcomes indicate that volumetric microsampling is generally accepted, technically feasible for home use, and capable of supporting adherence‐focused care models.

## Discussion

### State of the Evidence

This scoping review compiles current clinical evidence on volumetric microsampling for TDM and model‐based precision dosing. The 67 included studies enrolled more than 2000 participants across 18 countries and examined over 60 distinct drugs covering most major therapeutic classes in clinical pharmacology. The field is progressing from analytical demonstration to clinical implementation. Early studies focused primarily on method validation by comparison to vein reference samples,^48^, ^49^, ^89^ while recent research stresses at‐home sampling feasibility, patient acceptability, and routine outpatient implementation.^61^, ^64^, ^65^, ^75^ Technological advancements have driven the field, with new device formats creating opportunities later validated by clinical studies.

### Analytical Performance and Method Agreement

Across drug classes, agreement between VAMS‐derived and reference concentrations was generally strong. When Passing–Bablok regression and Bland–Altman analysis were reported (Table 3), median slopes approached unity, mean biases were typically within ±10%, and over 80% of paired samples fell within IATDMCT or ICH M10 acceptance windows. However, a considerable subset of studies did not report Passing–Bablok or association metrics in a standardized format; future reviews would benefit from harmonized reporting.

Two analytical topics are prominent. First, conversion between capillary whole‐blood VAMS and plasma reference values requires explicit documentation of the conversion method, if necessary. Empirical correction factors based on blood‐to‐plasma ratio, Passing–Bablok regression on patient samples, and recently developed hematocrit‐adjusted strategies each involve distinct assumptions. Few studies test their factor in a cohort separate from the one used to derive it, where this was done, for lacosamide, lamotrigine, and levetiracetam, the simple ratio approach held up better than regression. Clinically, the critical issue is whether bias is sufficient to alter dosing, a factor not always assessed. Hematocrit‐based correction has not been widely adopted and creates challenges, particularly in obtaining reliable capillary hematocrit measurements at home. A well‐designed fixed correction factor is often more practical. For tacrolimus and other blood‐targeted analytes, conversion is unnecessary. Second, analyte‐level hematocrit independence is observed for most drugs, but exceptions exist, such as abiraterone and cefepime at extreme hematocrit values.^40^, ^41^ Therefore, direct extrapolation across analytes is unsupported, and drug‐specific validation remains essential.

### Patient Acceptability and Home Sampling

Patient‐centric advantages were consistently observed, including the feasibility of home and self‐sampling.^24^, ^49^, ^58^, ^59^, ^61^, ^64^, ^71^, ^75^, ^87^, ^91^ Quantitative acceptability measures indicated a preference for microsampling in 80%‐100% of participants. Self‐collection feasibility ranged from clinic‐supervised to fully unsupervised home settings, including the postal mail‐back model used in transplant cohorts.^55^, ^61^ The study by Velghe et al in sub‐Saharan Africa demonstrates that patient‐centric approaches can be implemented in global‐health contexts lacking conventional TDM infrastructure.^79^ Among pediatric populations, reduced blood volume requirements, decreased pain, and the availability of non‐invasive salivary microsampling for selected analytes (perampanel^78^ and mycophenolic acid^52^) together address previous barriers to TDM in children. As each sample requires only microlitre volumes, microsampling facilitates serial collection, enabling estimation of full drug exposure (AUC) or the application of limited‐sampling strategies rather than dependence on a single trough value. This approach has been demonstrated for tacrolimus, including home‐based self‐collection, and is particularly advantageous for children who poorly tolerate repeated venipuncture.^63^, ^92^

### Drawbacks, Drug‐Specific Challenges, and Emerging Solutions

Several limitations of volumetric microsampling are consistently reported. First, certain analytes exhibit incomplete extraction or analyte‐specific bias. For example, abiraterone repeatedly failed to meet acceptance criteria,^40^ and a pediatric ICU study advised against VAMS for meropenem due to systematic underestimation in critically ill children.^83^ For most drugs, these challenges can be addressed through optimization; for others, they persist, necessitating an analyte‐specific approach. Second, variability in sampling technique during patient self‐collection can impact saturation and recovery. Factors such as fingerstick depth, application pressure, and timing of tip removal are significant, as demonstrated by Vethe et al in the differing success rates between Capitainer and Mitra under unsupervised conditions.^24^ Third, accurate hematocrit measurement for plasma‐target monitoring remains challenging. On‐tip hemoglobin or potassium‐based estimation, automated extraction (Carling et al^10^), and standardized conversion frameworks (Juan et al^44^) are progressively mitigating this issue.

Regulatory ambiguity remains a practical concern. While EMA, FDA, IATDMCT, and CLSI bioanalytical recommendations are frequently mentioned, no harmonized plan specifically addresses microsampling. The 2019 IATDMCT consensus guideline for tacrolimus monitoring includes references to microsampling and represents progress in this area^93^ and a wider microsampling guideline is currently under development by IATDMCT.^19^, ^36^ The reimbursement and accreditation pathways for VAMS‐based TDM vary across health systems and countries. Although cost‐effectiveness evidence is accumulating,^94^ it has not yet been formally evaluated in randomized trials. Modeling studies have begun to evaluate patient time, healthcare resources, and social perspectives, but published comparative effectiveness data remain limited.

### Strengths and Limitations of This Review

The strengths of this review include adherence to the PRISMA‐ScR checklist, prioritization of studies involving real patient cohorts and patient‐focused outcomes, and explicit documentation of analytical and clinical validation attributes across therapeutic areas. The limitations include restricting the review to English‐language publications. PubMed and Embase were both searched but Scopus, Web of Science, and grey literature were not searched, so some further studies are likely to remain unretrieved. Moreover, a formal backward citation search of the reference lists of included articles was not pre‐specified or conducted as a separate source‐identification method. In line with scoping review methodology, no formal critical appraisal was conducted. Heterogeneity in study design, analyte, device, and outcome reporting precluded quantitative synthesis. This review spans 2014, when VAMS was commercially introduced, to February 2026. Additionally, healthy‐volunteer pharmacokinetic studies were not categorically excluded if they assessed a patient‐centric parameter (e.g., the periodontitis amoxicillin study^84^), and this decision is documented in the data extraction form in the Supplemental file 1.

### Future Directions

This report identified five key priorities. First, prospective multicenter validation studies should compare MIPD using microsampled data with standard‐of‐care venous TDM, assessing both analytical agreement and clinically meaningful outcomes, such as dose changes, target attainment, and pharmacodynamic outcomes (such as transplant rejection rates and seizure frequency). Second, harmonized validation guidance customized to microsampling, including hematocrit testing, conversion factor reporting, and stability acceptance criteria, is needed and is currently being developed by IATDMCT; the tacrolimus consensus guideline^19^, ^93^ serves as a model. Third, integration with electronic health records, MIPD platforms, and clinical decision‐support tools is necessary to translate microsampled concentration data into actionable bedside recommendations. Fourth, systematic expansion into therapeutic proteins and biologics, as demonstrated by the Chi et al monoclonal antibody platform^74^ and the Soenen et al adalimumab study,^91^ is warranted given the increasing impact of biologics in pharmacotherapy. Fifth, formal cost‐effectiveness analyses that incorporate patient, healthcare‐resource, and societal perspectives are required to inform payer and policy decisions.

## Conclusion

Volumetric microsampling complies with EMA, FDA, IATDMCT, and CLSI bioanalytical standards in clinical patient populations, thereby supporting patient‐centered TDM and MIPD in clinical pharmacology. While the majority of experience has been gained in solid‐organ transplantation and immunosuppressive therapy, the technique is increasingly applied in oncology, neurology, infectious diseases, pediatrics, and, more recently, in therapeutic monoclonal antibody therapy. Where assessed, patients generally consider the process acceptable, and home‐based self‐sampling demonstrates feasibility across diverse populations and settings. Analytical performance aligns with established guidelines. Ongoing challenges, including drug stability, plasma conversion, and sampling variability, are manageable for most drugs. Certain analyte‐specific issues may persist, and these challenges are being addressed through improved validation, automation, and standardized conversion methods. Future research on MIPD validation, electronic health record integration, and cost‐effectiveness analysis will further inform the implementation of decentralized, microsampling‐based personalized pharmacotherapy.

## Author Contributions

**Hari Prabhath Tummala**: Conception; design; data acquisition; data analysis and interpretation; original draft; writing—review and editing. **Manjunath P. Pai**: Conception; design; data analysis and interpretation; supervision; writing—review and editing. All other authors participated in the design and writing—review and editing.

## Funding

The authors have nothing to report.

## Conflicts of Interest

None of the authors report relevant conflicts of interest with microsampling device makers.

## Supporting information



Supporting Information

## Data Availability

Data sharing not applicable to this article as no datasets were generated or analyzed during the current study.
