# Supplementary material for: Volumetric Microsampling for Patient‐Centric Therapeutic Drug Monitoring in Clinical Pharmacology: A Scoping Review
Source: J Clin Pharmacol. 2026 Sep 4;66(9):e70290. doi: 10.1002/jcph.70290 (PMC13543046; doi:10.1002/jcph.70290)
Supplement: Supplementary file 1 [file JCPH-66-0-s001.docx]

Search Strategy:

PubMed

1. **("volumetric absorptive micro sampling") OR ("VAMS") OR (mitra device) OR (tasso) OR ("qDBS")**
2. ("therapeutic drug monitoring") OR ("TDM") OR (dose selection) OR (dose adjustment) OR ("drug monitoring"[MeSH])
3. **#1 AND #2**

**Embase**

1. **('volumetric absorptive micro sampling' OR 'VAMS' OR 'mitra device' OR 'tasso' OR 'qDBS')**
2. **('therapeutic drug monitoring' OR 'TDM' OR 'dose selection' OR 'dose adjustment' OR 'drug monitoring'/exp)**
3. **#1 AND #2**
